# Supplementary material for: Rigid monoclonal antibodies improve detection of SARS-CoV-2 nucleocapsid protein
Source: bioRxiv. 2021 Jan 13:2021.01.13.426597. Preprint. [Version 1] doi: 10.1101/2021.01.13.426597 (PMC7814821; doi:10.1101/2021.01.13.426597)
Supplement: Supplement 1 [file media-1.pdf]

Supplemental material: Rigid monoclonal antibodies improve detection of SARS-CoV-2 nucleocapsid protein

Curtis D. Hodge, Daniel J. Rosenberg, Mateusz Wilamowski, Andrzej Joachimiak, Greg L. Hura, Michal Hammel

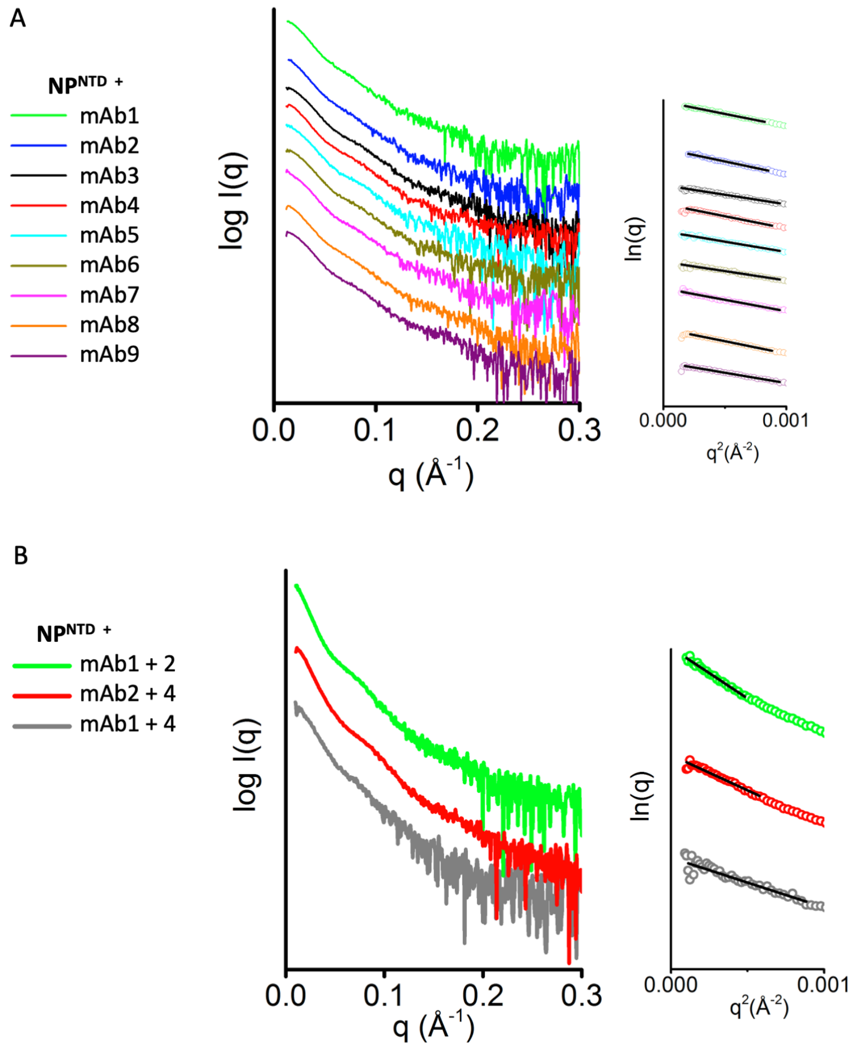

Supplementary Figure 1. SAXS profiles.
(A) Experimental SAXS curves of all tested mAb + NP^{NTD} samples (colored as indicated). Right panel: Corresponding Guinier plot with q*Rg <1.5 limit. (B) Experimental SAXS curves for the main SEC peak of mAb1-2-NP^{NTD} (green), mAb2-4-NP^{NTD} (red), mAb1-4-NP^{NTD} (gray). (A-B) Guinier plots were used to determine Rg values used in Figure 1B-inset and listed in Table 1.

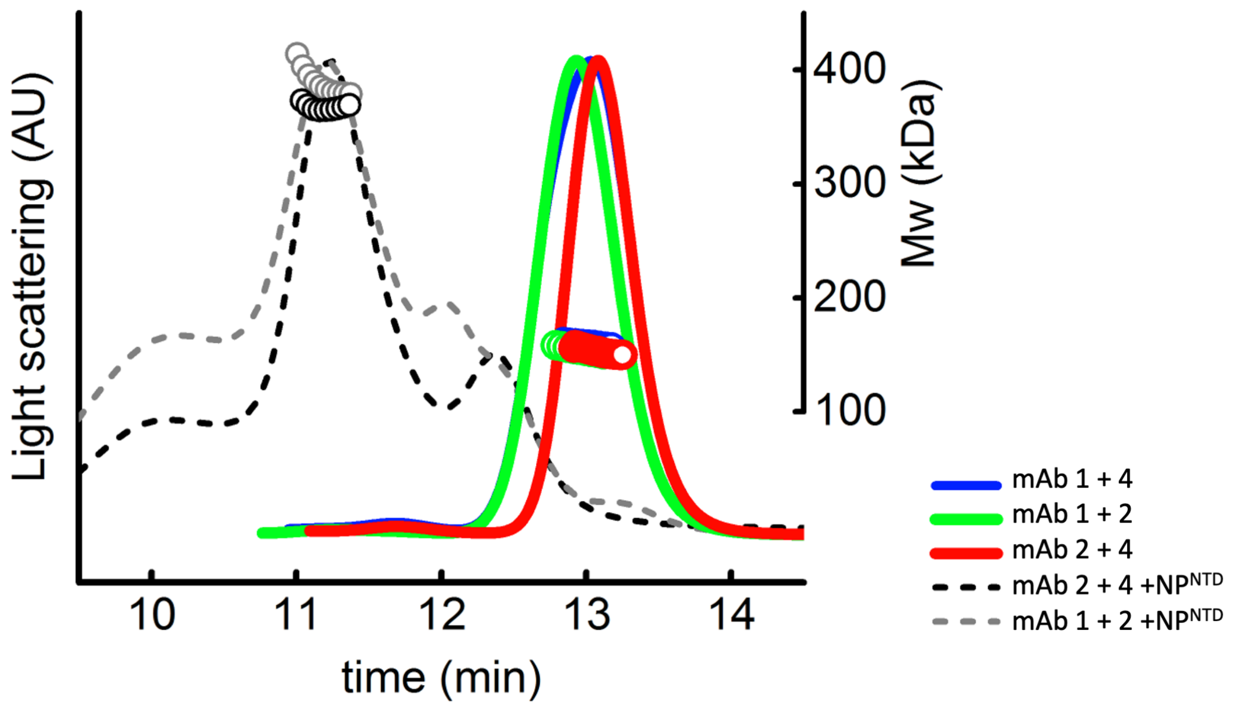

Supplementary Figure 2. mAb pairs do not form a large assembly in absence of NP^{NTD}.

SEC elution profile (lines) and MALS-determined molecular weight (circles) across the SEC peak for mAb1+4, mAb1+2, mAb2+4 in absence of NP^{NTD} (in comparison to the mAb1+2, mAb2+4 in presence of NP^{NTD} (colored as indicated).

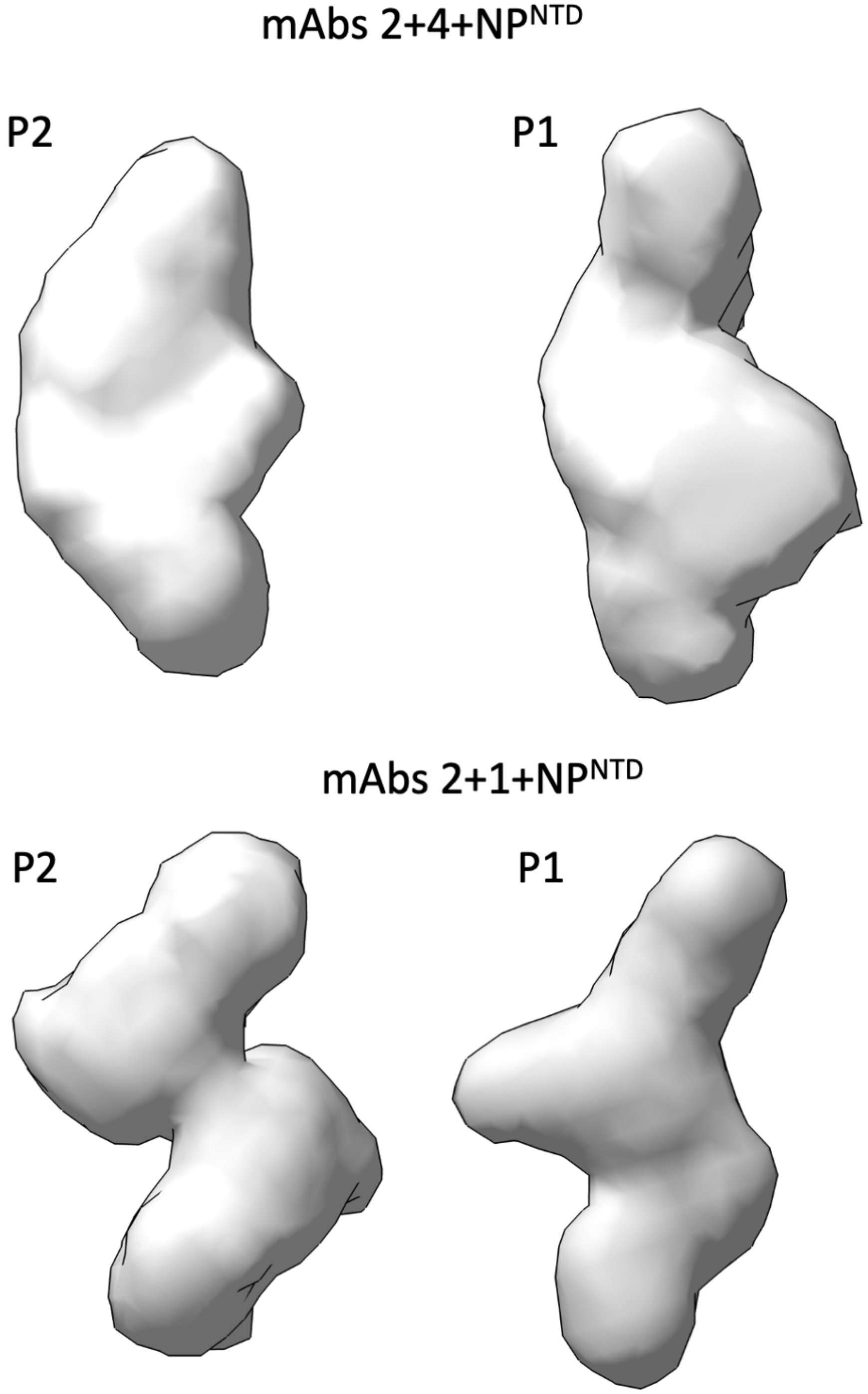

Supplementary Figure 3. Average SAXS envelopes. Comparison of average SAXS envelopes obtained for mAb2-4-NP^{NTD}, and mAb1-2-NP^{NTD} complexes reconstructed using a P2 (left) and P1 (right) symmetry operator.

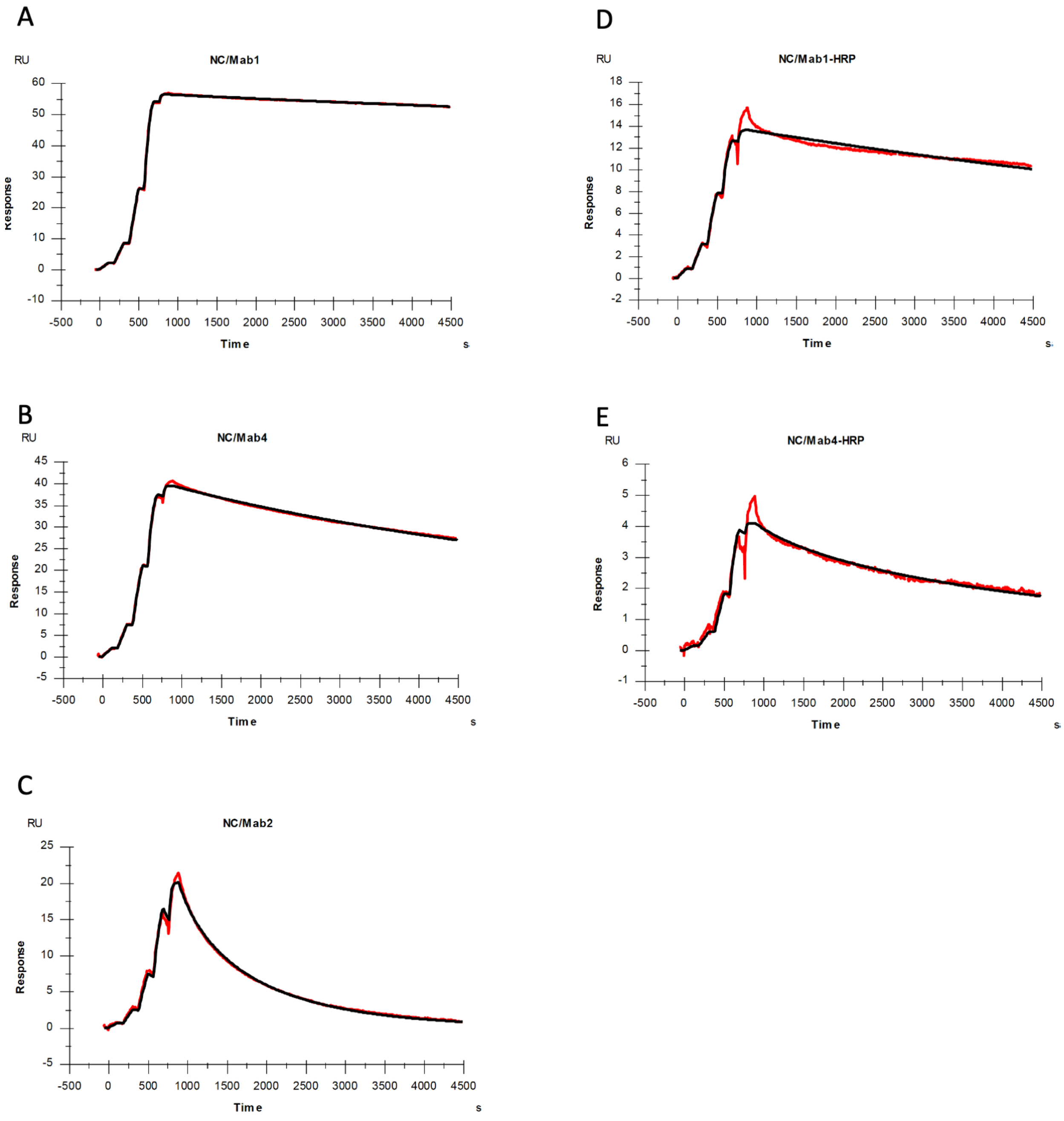

Supplementary Figure 4. Binding kinetics of NP^{NTD} with the mAb 1, 1-HRP, 2, 4, and 4-HRP by surface plasmon resonance (SPR).

NP^{NTD} at five concentrations (0.074nM, .22nM, .67nM, 2nM, 6nM) were tested using single-cycle kinetics with immobilized , mAb1 (A), mAb4 (B), mAb2 (C), mAb1-HRP (D), and mAb4-HRP (E).

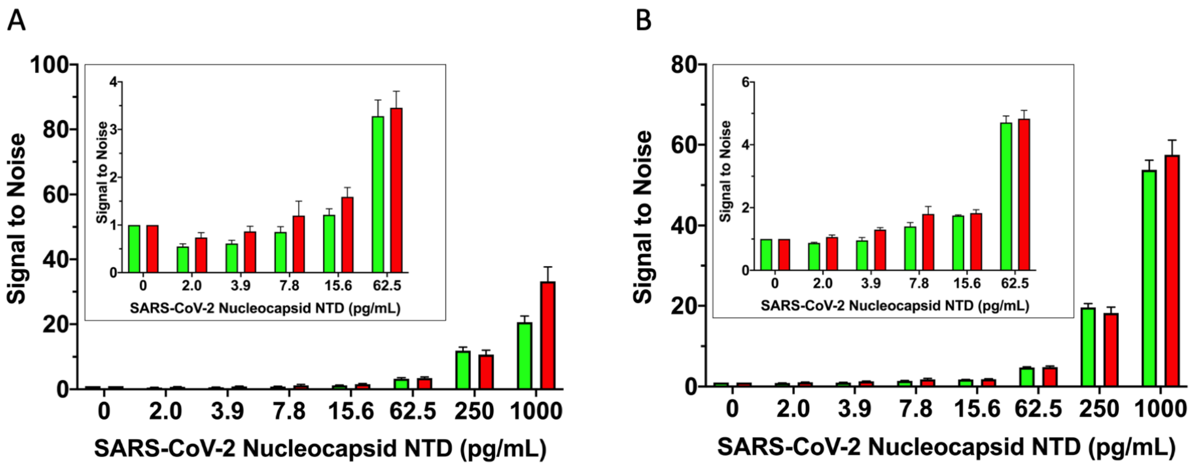

Supplementary Figure 5. Standard ELISA control protocol shows no boost.
(A) A standard ELISA where the plate was washed prior to addition of the detection HRP-conjugated mAbs (1-HRP in green, 4-HRP in red), for a twenty minute incubation. (B) A repeat of the experiment shown in A, except the mAb-HRPs had a longer incubation time of 1.5 hrs. In both experiments, no boost is observed, regardless of the mAb-HRP incubation time.

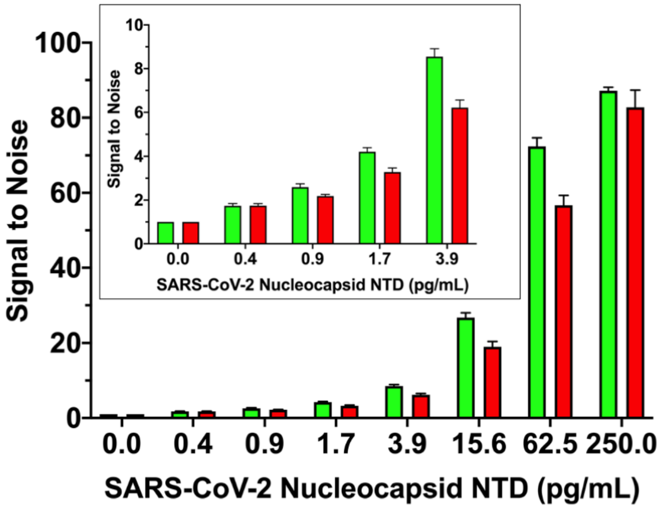

Supplementary Figure 6. The addition of virion lysing triton X-100 increases the LOD of the modified ELISA, diminishing the signal's boost.

(A) A modified ELISA where the detection HRP-conjugated mAbs (1-HRP in green, 4-HRP in red) are added directly on top of the samples during the NP^{NTD} capture incubation period, and free (non-plate-bound) mAb2 is “spiked” into the detection HRP-conjugated mAb solutions before their addition on top of the samples. The NP^{NTD} samples were diluted in PBS pH 7.4 plus 0.5% triton X-100.

Antibody	Association rate constant, k_a ($M^{-1}s^{-1}$)	Dissociation rate constant, k_d (s^{-1})	Dissociation equilibrium constant, K_D (pM)	Experimental Rmax (RU)	Theoretical Rmax (RU)	% Activity of mAb
**mAb 1**	**1.7×10^{7}**	**2.2×10^{-5}**	**1.3**	**56.4**	**65.0**	**87**
**mAb 1-HRP**	**7.6×10^{6}**	**8.5×10^{-5}**	**11.0**	**13.6**	**45.6**	**30**
**mAb 4**	**1.1×10^{7}**	**1.2×10^{-4}**	**11.0**	**39.4**	**43.4**	**91**
**mAb 4-HRP**	**1.7×10^{7}**	**4.7×10^{-4}**	**28.0**	**4.1**	**40.6**	**10**
**mAb 2**	**9.4×10^{6}**	**1.8×10^{-3}**	**190.0**	**20.7**	**39**	**53**

Supplementary Table 1.
